# Trends in Vegetation fires in South and Southeast Asian Countries

**DOI:** 10.1038/s41598-019-43940-x

**Published:** 2019-05-15

**Authors:** Krishna Prasad Vadrevu, Kristofer Lasko, Louis Giglio, Wilfrid Schroeder, Sumalika Biswas, Chris Justice

**Affiliations:** 1 NASA Marshall Space Flight Center, Huntsville, Alabama 35811 USA; 2 Geospatial Research Lab, US Army Corps of Engineers, Alexandria, Virginia 22315 USA; 3 NOAA NESDIS, College Park, Maryland 20740 USA; 4 Smithsonian Conservation Biology Institute, Front Royal, Virginia 22630 USA; 5 University of Maryland, College Park, Maryland 20742 USA

**Keywords:** Ecology, Natural hazards

## Abstract

We assessed the fire trends from Moderate Resolution Imaging Spectroradiometer (MODIS) (2003–2016) and Visible Infrared Imaging Radiometer Suite (VIIRS) (2012–2016) in South/Southeast Asia (S/SEA) at a country level and vegetation types. We also quantified the fire frequencies, anomalies and climate drivers. MODIS data suggested India, Pakistan, Indonesia and Myanmar as having the most fires. Also, the VIIRS-detected fires were higher than MODIS (AQUA and TERRA) by a factor of 7 and 5 in S/SEA. Thirty percent of S/SEA had recurrent fires with the most in Laos, Cambodia, Thailand, and Myanmar. Statistically-significant increasing fire trends were found for India (p = 0.004), Cambodia (p = 0.001), and Vietnam (p = 0.050) whereas Timor Leste (p = 0.004) had a decreasing trend. An increasing trend in fire radiative power (FRP) were found for Cambodia (p = 0.005), India (0.039), and Pakistan (0.06) and declining trend in Afghanistan (0.041). Fire trends from VIIRS were not significant due to limited duration of data. In S/SEA, fires in croplands were equally frequent as in forests, with increasing fires in India, Pakistan, and Vietnam. Specific to climate drivers, precipitation could explain more variations in fires than the temperature with stronger correlations in Southeast Asia than South Asia. Our results on fire statistics including spatial geography, variations, frequencies, anomalies, trends, and climate drivers can be useful for fire management in S/SEA countries.

## Introduction

Vegetation fires are a common phenomenon in many different regions of the world including South/Southeast Asia (S/SEA). Fuel type, topography, climate, weather, lightning, and other factors govern fire occurrence and spread^1–4^. Of the different natural factors, drought-induced fires due to El Niño-Southern Oscillation (ENSO) in southeast Asia and more specifically Indonesia are most common^5–7^. In addition to these natural factors, most of the fires in S/SEA are human initiated. For example, fire is used as a land clearing tool during the slash and burn agriculture in the Eastern Ghats and northeast India^4,8–10^, Chittagong hill tracts of Bangladesh^11^, Myanmar^12,13^, Sarawak in Malaysia^14^ Philippines^15^, Jambi, Sumatra and others in Indonesia^16,17^, northern Thailand^18^, northern Laos^19,20^, Cambodia^21^, and northern Vietnam^22^. Fires are also extensively used for clearing land for oil palm expansion in Indonesia^23,24^. In addition, most of the countries in S/SEA are agrarian where farmers use fire for the burning of agricultural residues to clear the land for the next crop^25–28^.

The impacts of fire on ecosystems vary as a function of severity and management^29^. Forest fires can result in the loss of biodiversity including the disruption of soil microbial processes during vegetation combustion and alter biogeochemical cycles^30,31^. Fires can create landscape disturbance resulting in a mosaic of burned and unburned forest patches, leaving complex heterogeneous patterns across the landscape^32^. The resulting landscape heterogeneity can further influence successional processes, which in turn may affect the spatial spread of subsequent fires^33^. In addition, vegetation fires can release large greenhouse gas emissions such as CO_2_, CO, NOx, CH_4_, non-methane hydrocarbons and other chemical species including aerosols impacting radiative budget, air quality and health at both local and regional scales^28,34–38^. Further, the pollutants can be transported over long distances impacting not only local climate, but also regional climate^39,40^. Considering these impacts, characterizing fires in different regions of the world is important, including in S/SEA countries. In particular, quantifying vegetation fire trends and anomalies can help in identifying countries where fires have been increasing or decreasing and subsequently relating the fire occurrences to driving factors such as climate, topography, vegetation and anthropogenic factors. An in-depth analysis of the type of vegetation burnt, fire intensities and also timing can help inform fire management at different spatial scales.

Specific to mapping and monitoring of fires and related vegetation changes, remote sensing technology has been playing a vital role over the past several decades^41^. Some of the most commonly used satellite fire data sets include active fire detection and burnt area mapping products. In addition to the Earth Observing System Moderate Resolution Imaging Spectroradiometer (MODIS) fire products available since 2002 (http://modis-fire.umd.edu/index.php), fire products from the Joint Polar Satellite System Visible Infrared Imaging Radiometer Suite (VIIRS) data have been readily available since 2012 (http://VIIRSfire.geog.umd.edu/). These satellite fire data records provide a unique opportunity to assess fire trends in different regions of the world.

Using the above satellite fire data, we address the following questions specific to vegetation fire trends in S/SEA countries: Are vegetation fires increasing or decreasing in Asian countries? Which of those countries show the highest vegetation fire concentration? Are the vegetation fire trends consistent between the MODIS and VIIRS data? How do fire trends vary across land cover types (agriculture, forests, grasslands, and shrublands?) How does fire radiative power (FRP), which is an indicator of fire intensity, vary across different vegetation types and what are the trends? We address these questions using both the MODIS and VIIRS fire datasets. Understanding the similarities and discrepancies between the MODIS and the VIIRS is important as their spatial resolutions are different. Also, the MODIS sensor on Aqua and Terra satellites are nearing the end of their lives^42^ thus, the potential of the other  sensors designed for their continuity should be explored. The results on the vegetation fire trends can help to address fire management and mitigation related issues including drivers in different countries.

## Datasets and Methods

### MODIS active fire Products

We used the latest collection 6 MODIS active fire products (MCD14ML) from the University of Maryland (http://modis-fire.umd.edu/index.php). The active fire dataset is at a 1 km nominal spatial resolution; however, it can detect flaming fires as small as 50 m^2^ under ideal observing conditions^41^. The MODIS fire product provides the latitude and longitude of the fire pixels, the date and time of the fire detection, FRP including confidence levels of fire detection. Specific to this study, we used fires with confidence greater than forty percent. The FRP parameter is a quantitative measure of radiant heat output commonly used to approximate fire intensity, which is proportional to its combustion rate and smoke emissions^43–45^.

### VIIRS I-band fire product

The first VIIRS instrument was launched in October 2011 aboard the Suomi-National Polar-orbiting Partnership (S-NPP) satellite. The VIIRS instrument carries two separate sets of multi-spectral channels providing global coverage at both 375 m and 750 m nominal resolutions every 12 h or less depending on the latitude. The VIIRS satellite incorporates fire-sensitive channels, including a dual-gain, high-saturation temperature 4 µm channel enabling active fire detection and characterization. Active fire products based on the 375 m (I-bands) and 750 m (M-bands) VIIRS data are currently being generated^46^ (https://viirsland.gsfc.nasa.gov/Products/NASA/FireESDR.html; https://www.star.nesdis.noaa.gov/jpss/fires.php). In this study, we specifically used the VIIRS 375 m active fire product (VNP14IMG) reprocessed by NASA. The algorithm for this product builds on the well-established MODIS Fire and Thermal Anomalies product using a contextual approach to detect thermal anomalies^41^. Due to its higher spatial resolution, the VNP14IMG active fire product captures more fire pixels than MODIS MCDML product^46^. The confidence information is given in the data at three different levels, i.e., low, nominal and high. We used nominal and high level sorted fire data in this study. Specific to the FRP, the VNP14IMG FRP is calculated through a combination of both VIIRS 375 m and 750 m data. The former is used to identify fire, cloud (solid blue), water (dashed blue), and valid background pixels. The co-located M13 channel radiance data (750 m) coinciding with fire pixel and valid background pixels are used in the FRP calculation (https://viirsland.gsfc.nasa.gov/PDF/VIIRS_activefire_375m_ATBD.pdf).

### Fire radiative power products

FRP is the rate of radiative fire energy released per unit time, measured in megawatts^43^. Fire radiative energy (FRE) is therefore FRP integrated over time and space, with units of mega joules (MJ). For the VNP14IMG product, 375 m mid-IR (I4) radiance data are not used for FRP retrieval due to frequent saturation/folding and incorrect assignment of quality flags during L1B onboard data aggregation^46^. Instead, FRP is retrieved using co-located dual-gain mid-IR M13 channel (750 m) for all fire pixels detected using the 375 m data. Both MODIS and VIIRS FRP (MW) retrievals are use the^47^ approach in which FRP is approximated as,

$$FRP\approx \frac{{A}_{pix}\sigma }{a\,{\tau }_{4}}({L}_{4}-{\bar{L}}_{4})$$

where L4 is the 4-μm radiance of the fire pixel, ${\bar{L}}_{4}$ is the 4-μm background radiance, Apix is the area of the pixel (which varies as a function of scan angle), σ is the Stefan-Boltzmann constant (5.6704 × 10^−8^ W m^−2^ K^−4^), τ_4_ is the atmospheric transmittance of the 4-μm channel, and a is a sensor-specific empirical constant (a = 2.88 × 10^−9^ W m^−2^ sr^−1^ μm^−1^ K^−4^ for VIIRS, and a = 3.0 × 10^−9^ W m^−2^ sr^−1^ μm^−1^ K^−4^ for MODIS, when radiance is expressed in units of Wm^–2^ sr^–1^ μm^–1^).

FRP measurements have been previously related to the amount of biomass burnt^47^, the strength of fires^48^ and aerosol emissions^44,49,50^. In this study, we used FRP as an indicator of the intensity of fires. We used trend analysis to infer if the fire intensities increased or decreased over a period in different ecosystems and countries. Details about the trend analysis are given below.

### Vegetation fire analysis

We used the latest European Space Agency Climate Change Initiative 300 m land cover product version 2.0.7 derived from a time series of MEdium Resolution Imaging Spectrometer (MERIS) surface reflectance data^51,52^. We acquired the product from the ESA-CCI website for 2012–2015 (http://maps.elie.ucl.ac.be/CCI/viewer/). Because no data are available beyond 2015, we used 2015 data for 2016 in order to match it to the 2012–2016 active fire record analyzed in this study. Subsequently, we simplified the land cover classes creating five broad groups including: croplands (classes 10, 20, 30), forest (classes 12, 40–100, 160, 170), shrubland(classes 11, 110, 120, 121, 122, 180), grassland/sparsely vegetated (130, 140, 150, 151, 152, 153, 200, 201, 202), and other (190, 201, 220). Specific to Indonesia, the forests also include peatlands. Land cover information was then extracted for each corresponding MODIS and VIIRS active fire datasets and used in the trend analysis.

### Trend analysis

We used both the MODIS Aqua and Terra (2003–2016) and VIIRS fire data (2012–2016) and vegetation types retrieved from the MERIS data to assess the fire trends in S/SEA countries. For the trend analysis, we applied the non-parametric seasonal Mann-Kendall statistical test, which doesn’t require a normally-distributed sample. The method is less influenced by outliers since its calculation is based on the sign of the differences, and not directly on the variable values. Another advantage of this test is its low sensitivity to abrupt breaks due to in-homogenous time series^53^. Sen’s slope estimator, which gives the trend magnitude, complemented our analysis^54^. The seasonal trend test is used since it assesses the data for different seasons (individual months in our case) over a period.

The Mann Kendall test consists of comparing each value of the data series with the subsequent values, calculating the number of times that the remaining terms are greater than the analyzed value^55^. The nonparametric test is used to analyze the existence of a monotonically increasing or decreasing trend. According to this test, the null hypothesis H0 states that the deseasonalized data (x1, …, xn) is a sample of n independent and identically distributed random variables. The alternative hypothesis H1 of a two-sided test is that the distributions of xk and xj are not identical for all k, j ≤ n with k ≠ j. The test statistic S, which has mean zero and variance is given as,

$$S=\sum _{k=1}^{n-1}\sum _{j=k+1}^{n}sgn({x}_{i}-{x}_{j})$$

with *sgn* denoting the signum function, and where x_j_ and x_k_ are values (annual/seasonal/monthly) in years *j* and *k* respectively; *n* is the size of the data series. The variance of S, denoted by [VAR (S)], assumes the value of 1 when *xj* − *xk* > 0; 0 when *xj* − *xk* = 0; and −1 when *xj* − *xk* < 0, and is defined as,

$$Var(S)=\frac{[n(n-1)(2n+5)-{\sum }_{t}t(t-1)(2t+5)]}{18}$$

The notation *t* is the extent of any given tie, and denotes the summation over all ties. In cases, where sample size n > 10, the standard normal variable Z is computed as,

$$Z=\{\begin{array}{ll}\frac{S-1}{\sqrt{Var(S)}} & if\,S > 0\\ 0 & if\,S=0\\ \frac{S+1}{\sqrt{Var(S)}} & \,if\,S < 0\end{array}$$

Positive values of Z indicate increasing trends, while negative values of Z show decreasing trends. When testing either increasing or decreasing monotonic trends at the significance level, the null hypothesis was rejected for an absolute value of Z greater than Z_1- α/2_, obtained from the standard normal cumulative distribution tables.

In addition, we also calculated the Sen’s^54^ slope. The slope estimates of N pairs of data are first computed by,

$$\begin{array}{ll}{Q}_{i}=\,\frac{{x}_{j}-{x}_{k}}{j-k} & {\rm{for}}\,i=1,\,\ldots \mathrm{..},\,N\end{array}$$

where x_j_ and x_k_ are data values at times j and k (j > k), respectively. The median of the N values of Q_i_ represents the Sen’s estimator of slope. If N is odd, the Sen’s estimator is computed as

$${Q}_{med}={Q}_{[(n+1)/2]}$$

If N is even, it is computed as,

$${Q}_{med}=\,\frac{1}{2}({Q}_{[N/2]}+{Q}_{[(N+2/2)]})$$

The Q_med_ is tested with a two-sided test at the 100 (1 − α) % confidence interval and the true slope may be obtained with the non-parametric test^56,57^. The confidence interval (*C*_*α*_) is given as,

$${C}_{\alpha }={Z}_{1-\alpha /2}\sqrt{Var(S)}$$

where Var (S) is given as equation (3)

### Fire standardized anomalies

Using the MODIS active fire data from 2003–2016, we computed the standardized anomalies (SD) for different countries as,

$$SD=(\frac{X-\mu }{\sigma })$$

where *X* is the active fire count for specific year and country, and $\mu$ and $\sigma$ are the respective mean and standard deviation of active fire counts from 2003–2016. Standardized anomalies were useful to delineate specific years where fires were prevalent.

### Climate datasets

We also assessed the long-term relationship between the fires and the corresponding precipitation and temperature datasets. Specifically, we used the 4.01 release of the Climate Research Unit (CRU) CY dataset available from 1901–2016. The dataset consists of country averages at a monthly, seasonal and annual frequency, for ten different variables. For the study, we used a mean monthly temperature and precipitation data from CRU and mean monthly fires from MODIS (2003–2016) for different countries to assess the fire-climate relationships.

## Results and Discussion

### MODIS fires in different countries

**Figure 1:**
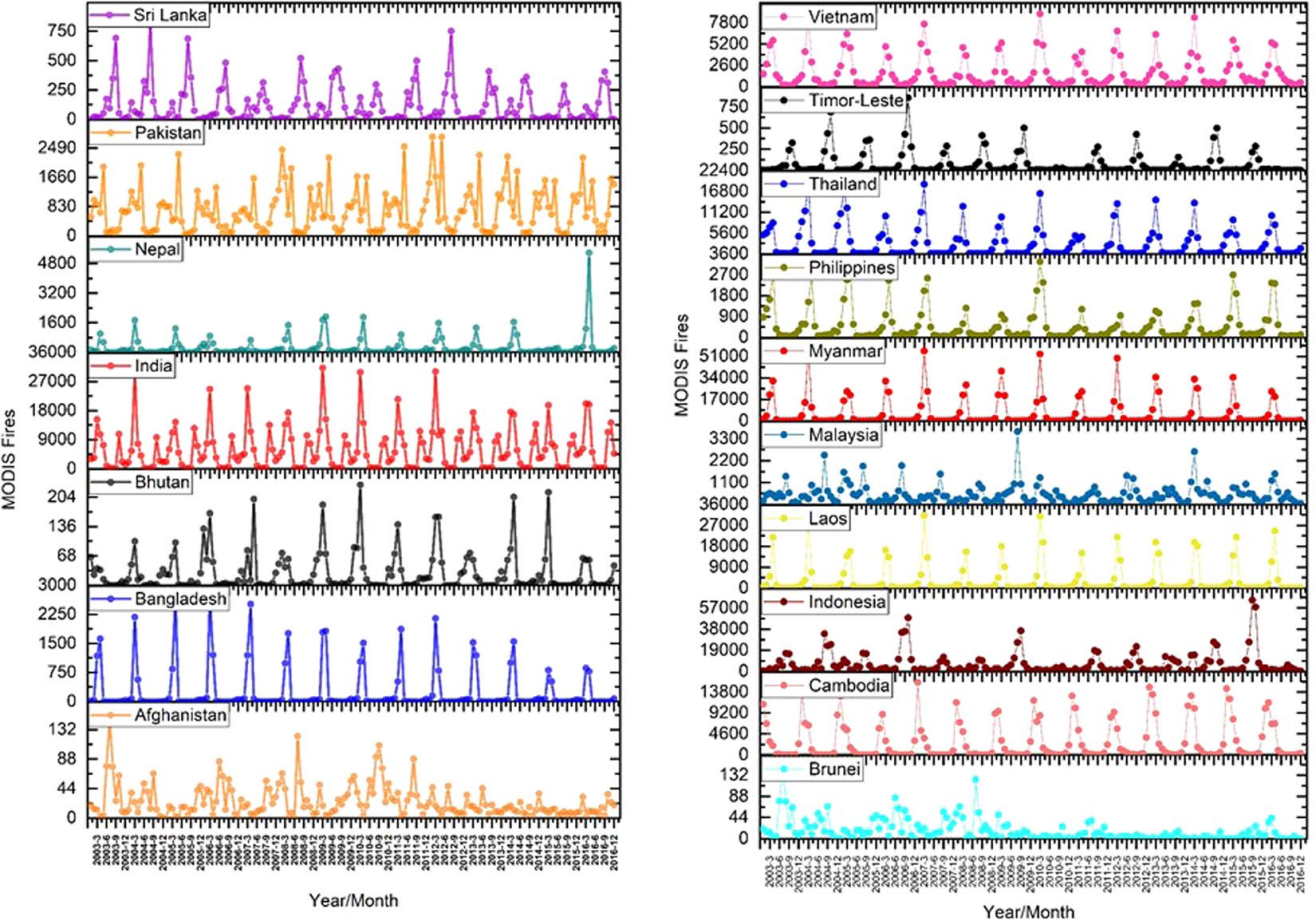
(**a**,**b**) Trends in vegetation fires in South Asian countries retrieved using MODIS Aqua and Terra combined data (2003–2016). A relatively higher number of fires can be seen for India in South Asia and Indonesia and Myanmar in Southeast Asia every year.

Results from averaging the MODIS fire data from 2003–2016 showed India with the highest number of annual fire counts of 73836 followed by Pakistan (8761), Nepal (3049), Bangladesh (3029) and the lowest in Afghanistan (298). Averaging the VIIRS fire data from 2012–2016 suggested India having the highest number of annual fire counts of 551649, Pakistan (53998), Nepal (32018), Bangladesh (10972), Sri Lanka (9185), Bhutan (1411), and least for Afghanistan (1075) (Fig. 1).

In Southeast Asia, averaging the MODIS fire data from 2003–2016 suggested highest fire counts for Indonesia (79476), followed by Myanmar (70084), Laos (39666) and the lowest for Brunei (75). VIIRS detected fires (2012–2016) showed the highest number of fires for Indonesia (412080) followed by Myanmar (368533), Cambodia (199646), others and least for Singapore (32).

**Figure 2:**
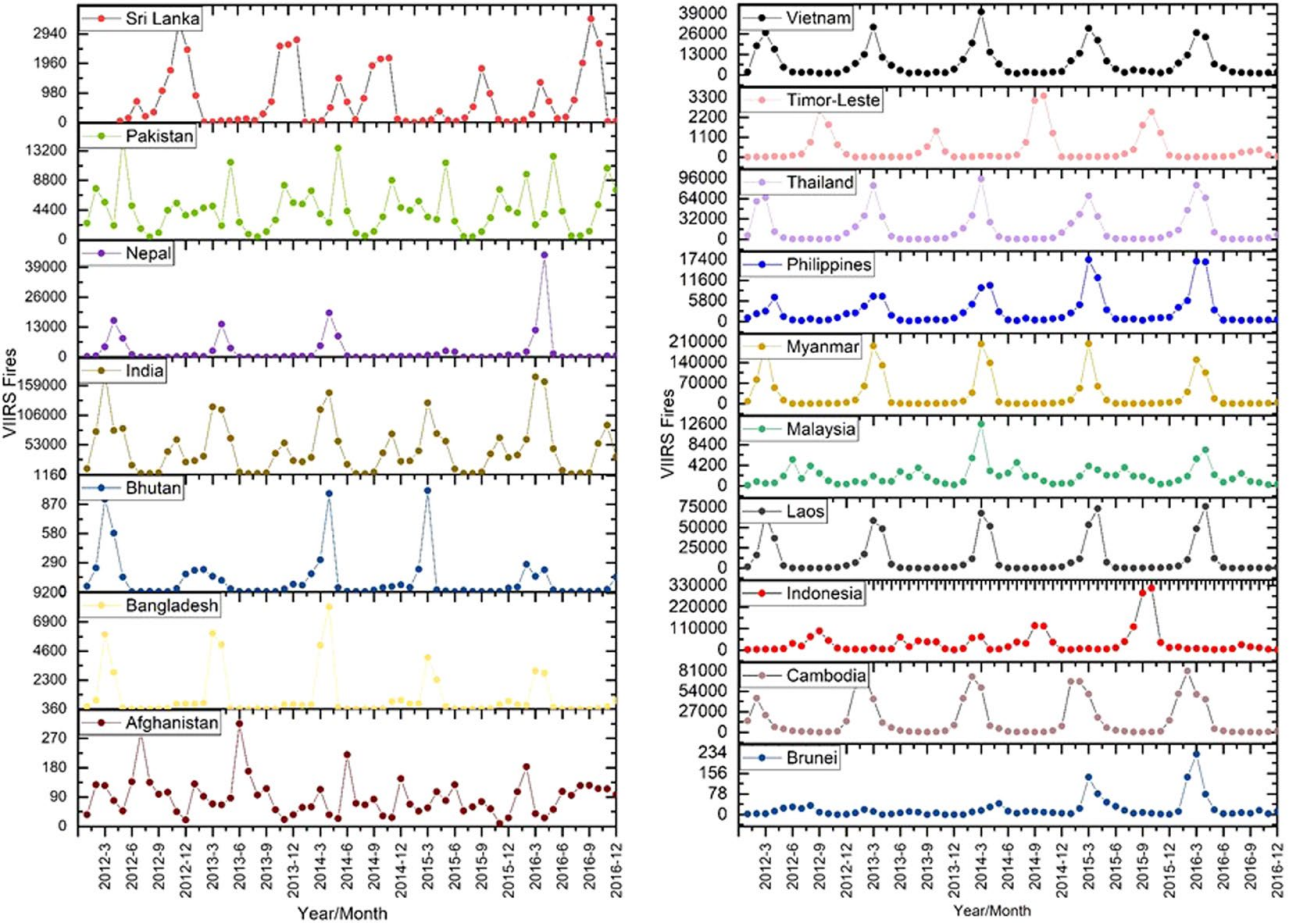
(**a**,**b**) Trends in vegetation fires in South Asian countries retrieved using VIIRS 375 I-band data (2012–2016). A relatively higher number of fires can be seen for India in South Asia and Indonesia and Myanmar in Southeast Asia every year.

Trends in vegetation fires in South and Southeast Asia countries retrieved using VIIRS 375 I-band data (2012–2016) are shown in Fig. 2. Table 1 highlights the differences in MODIS (Aqua and Terra combined) and VIIRS active fires. Of the different countries in South Asia, VIIRs detected fires were 17.71 higher in Nepal than the MODIS. This is a significant improvement considering the hilly terrain in Nepal. In overall, the VIIRS detected fires were higher than MODIS by a factor of 7.2 in South Asian countries and 5.12 factor higher in Southeast Asian countries. The higher detection of fires by VIIRS can be primarily attributed to its finer spatial resolution and higher pixel fidelity along the swath due to pixel aggregation scheme compared to MODIS^46^. The results can have significant implications concerning increased smoke release and contribution to GHG emissions and aerosols.

**Table 1:** MODIS Fires (Aqua and Terra Combined) in comparison to VIIRS.

Country	MODIS	VIIRS	Factor
Afghanistan	298	1075	3.60
Bangladesh	3029	10972	3.62
Bhutan	347	1411	4.07
India	73836	551649	7.47
Nepal	3049	53998	17.71
Pakistan	8761	32018	3.65
Sri Lanka	1417	9185	6.48
**South Asia Average**	**12962**	**94330**	**7.28**
Brunei	75	253	3.37
Cambodia	32806	199646	6.09
Indonesia	79476	412080	5.18
Laos	39666	142354	3.59
Malaysia	6068	24470	4.03
Myanmar	70084	368533	5.26
Philippines	5977	35038	5.86
Singapore	7	32	4.63
Thailand	32533	194262	5.97
Timor-Leste	857	5118	5.97
Vietnam	20346	90917	4.47
**Southeast Asia Average**	**26172**	**133882**	**5.12**

Fire data from 2003–2016 for MODIS and 2012–2016 for VIIRS has been averaged for different countries. VIIRS detected fires were much higher than MODIS as shown in the factor column.

### MODIS fire return frequency

**Figure 3:**
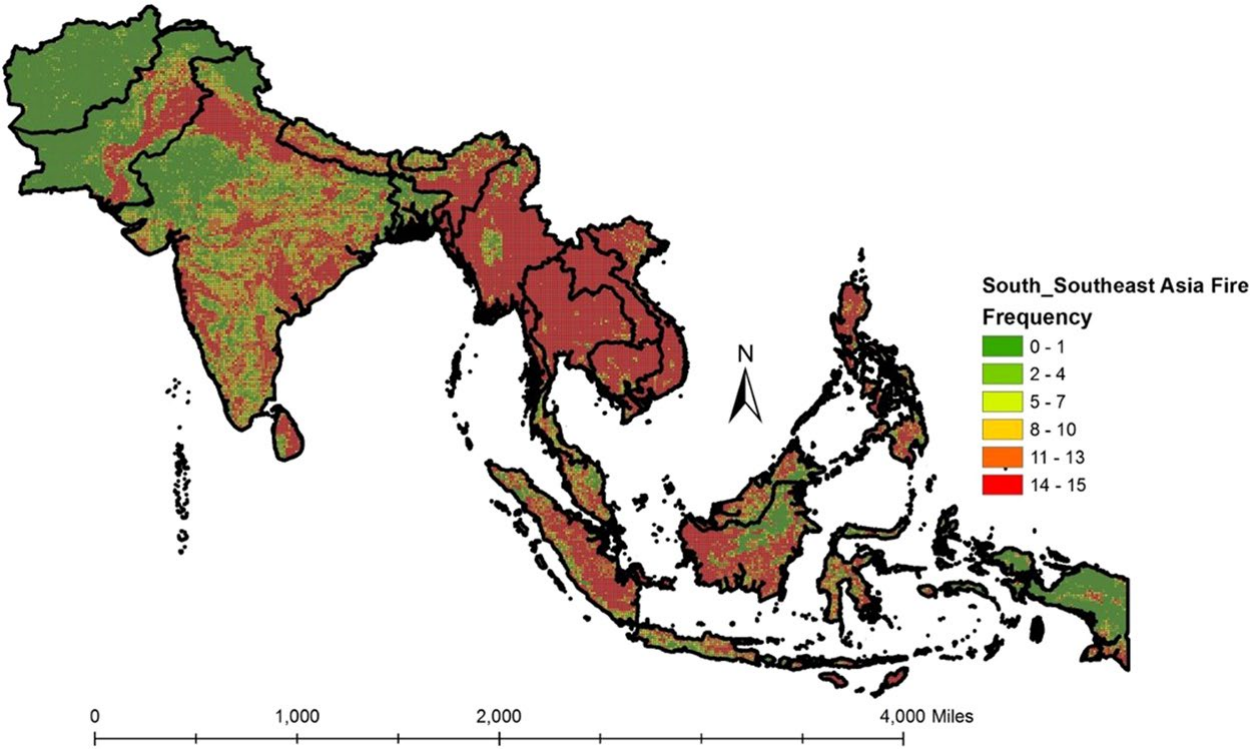
Fire frequencies in South and Southeast Asia at 10-minute grid intervals. The scale varies from 0–15, indicating the lowest value of 0 with grid cells without fires and a maximum value of 15 with the grid cells having fires every year during the fifteen years (2003–2017) time period. Relatively higher fire frequencies can be found for Myanmar, Thailand, Cambodia, and Laos in Southeast Asia.

Spatially-gridded fire return frequencies at 10-minute grid intervals for the entire S/SEA from 2003–2017 are shown in Fig. 3. The scale varies from 0–15, indicating the lowest value of 0 with cells without fires during fifteen-years (2003–2016) and a maximum value of 15 for grid cells with fires every year. Table 2 highlights the percentage of 10-minute grid cells impacted by fires in different S/SEA countries. Thus, for example, of the total cells of 9555 grid cells in India, 1701 cells (17.80%) were impacted by fires every year (i.e., frequency = 15). Of the different countries, Laos, Cambodia, Thailand, and Myanmar had the highest percentage of recurring fires. Further, 30% of grid cells in S/SEA countries (Table 3) had fires occurring every year.

**Table 2:** MODIS Fires (Aqua and Terra combined) at 10-minute grid intervals depicting cells impacted by fires every year over a fifteen year time period (2003–2017).

Country	Total 10 m Grid cells	Cells with a fire frequency of 15	% of grid cells
India	9555	1701	17.802
Bangladesh	323	37	11.455
Bhutan	101	7	6.931
Myanmar	1849	1389	75.122
Cambodia	471	371	78.769
Indonesia	4572	974	21.304
Laos	575	551	95.826
Malaysia	785	124	15.796
Nepal	410	64	15.610
Pakistan	2747	397	14.452
Philippines	577	208	36.049
Srilanka	178	80	44.944
Thailand	1345	1009	75.019
Vietnam	792	494	62.374
Total	24280	7406	30.502

Thirty percent of 10-minute grid cells in south/southeast Asia had recurrent fires (2003–2017).

**Table 3:** Trends in MODIS Fires (Aqua and Terra Combined) for different Countries (2003–2016).

Country	FC Seasonal Kendall Test	FC Sens slope Estimator	FRP Seasonal Kendall Test	FRP Sens Slope Estimator
Cambodia	426 (0.001)	19.733	340 (0.005)	307.4
Indonesia	28 (0.8)	9.92	−44.0 (0.695)	−449
Laos	15 (0.773)	0	22 (0.688)	22.0
Malaysia	−59 (0.455)	−3.0	−42.0 (0.515)	−58.19
Maldives	−86 (0.251)	0	−112 (0.183)	−0.743
Myanmar	23 (0.736)	0.127	−14 (0.817)	−2.906
Philippines	−75 (0.435)	−1.917	−82 (0.429)	−39
Thailand	−69 (0.224)	−2.667	−100 (0.254)	−48.86
Timor Leste	−163 (0.004)	−0.40	−169 (0.99)	−6.844
Vietnam	126 (0.050)	12.8	30 (0.597)	68.64
Afghanistan	−103 (0.214)	−0.5	−116 (0.041)	−15.77
Bangladesh	145 (0.078)	0.222	57 (0.446)	0.747
Bhutan	−22.0 (0.685)	0.724	−22.0 (0.638)	0
India	218 (0.004)	20.857	102 (0.039)	131.49
Nepal	21 (0.714)	0	−12.0 (0.841)	0
Pakistan	136 (0.074)	6.417	130 (0.066)	116.09
Sri Lanka	−153 (0.072)	−0.50	−166 (0.049)	−10.54

### MODIS fire and FRP trends in different countries

Trends in MODIS fires for different countries are given in Table 3. Of the different countries, increasing trend in fires were found for India (p = 0.004), Cambodia (p = 0.001), and Vietnam (p = 0.050) and decreasing trend for Timor Leste (p = 0.004). Thiel’s Sen’s slope which indicates the magnitude in trend was relatively high for countries with increasing fires, i.e., India, Vietnam, and Cambodia. Pakistan and Bangladesh had higher p-values of 0.074 and 0.078 for fires with a positive slope. In case of FRP, Afghanistan and Sri Lanka had a negative slope value of 0.04 and 0.049 whereas Cambodia, India, and Pakistan had a positive slope with highly significant (*p*) values (Table 3). These results suggest increased fires as well as FRP for India and Cambodia as depicted in highly significant p values in M-K test as well as Sen’s slope estimator.

### MODIS fires in different land cover categories

**Figure 4:**
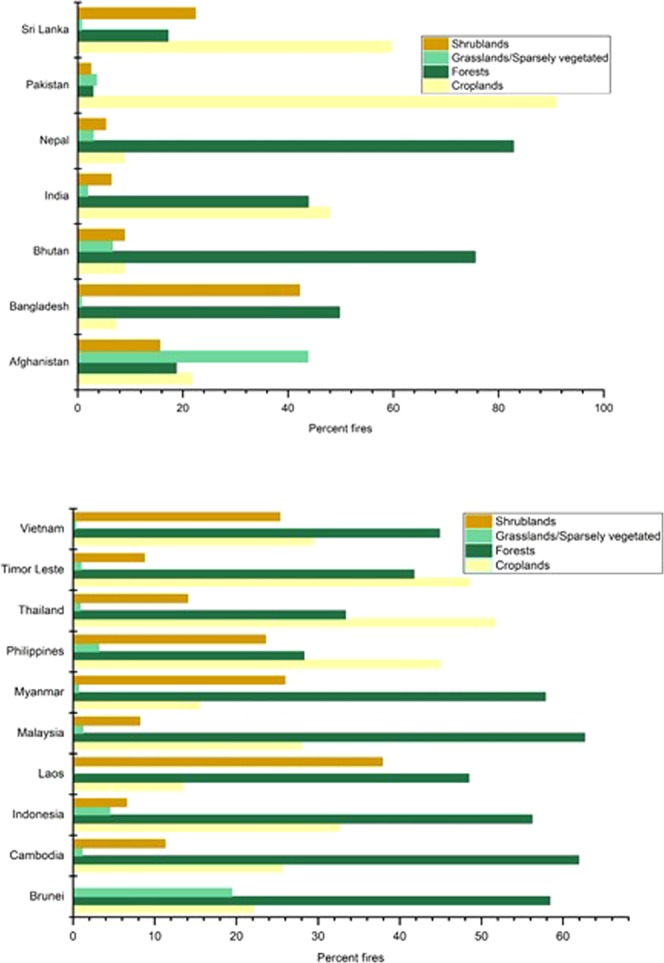
(**a**,**b**) MODIS (Aqua and Terra) retrieved fires in different land cover types in South Asia and Southeast Asian countries. MERIS (300 m) data has been used to retrieve land cover types in different countries. Fires in croplands and forests dominate different countries.

Using MERIS data (300 m), MODIS fire data has been partitioned into different land cover types to assess the major land cover types with the highest percentage of fires. In South Asia, Pakistan (91%), Sri Lanka (59.70%), and India (47.92%) had the highest percentage of fires in croplands mainly attributed to agricultural residue burning. Most of the agricultural fires in India and Pakistan are due to rice-wheat and sugarcane crop residue burning in the Indo-Ganges region^26,34,58^. In Sri Lanka, information on agricultural residue burning is meagre however one of the recent studies suggests Rice straw as the most commonly burnt residue^59^. In contrast, Nepal (82.84%) and Bhutan (75.56%) had the highest percentage of fires in forest land cover types which are mostly human initiated. The primary causes include agricultural fires spreading from farms to forests, abandoned cooking fires, carelessness due to smokers, firewood collectors^60^. In Afghanistan, 43.75% of total fires were in grasslands/sparsely vegetated category mostly in the Hindu Kush alpine meadow as well as the Ghorat-Hazarajat alpine meadow. In Bangladesh (42.17%) and Sri Lanka (22.39%) had relatively higher percent fires were found in shrublands suggesting degraded nature of forests which are highly vulnerable to fires (Fig. 4a,b).

In Southeast Asia, Thailand (51.75%), followed by Timor Leste (48.75%) and the Philippines (44.99%) had the highest percentage of fires in croplands due to rice, maize and sugarcane agricultural residue burning^27,61,62^, whereas Malaysia (62.65%), Cambodia (61.94%), Brunei (58.37%) and Myanmar (57.82%) had the highest percentage of fires in forests mainly due to slash and burn agriculture, and timber harvesting. Brunei also had the highest percent of fires in grassland/sparsely vegetated category, whereas, Laos (37.88%), Myanmar (25.95%), Vietnam (25.33%) and the Philippines (23.58%) had the highest percentage of fires in the shrublands category.

### MODIS fire and FRP trends in different land cover types

We also assessed the fire and FRP trends in different land cover types (Supplementary Material). MERIS (300 m) data has been used for vegetation partition into four major categories, i.e., croplands, forests, grasslands, and shrublands. Increasing fire trends in croplands are found for India, Pakistan and Vietnam attributed to agricultural residue burning whereas decreasing trend has been found for Malaysia. In the case of forests, except Cambodia which had a positive trend, Afghanistan, Thailand, Pakistan, and Sri Lanka showed a negative trend. India, Pakistan, Indonesia, and Cambodia showed a positive fire trend in grasslands, whereas, Maldives, Nepal and Sri Lanka showed a negative trend. Further, in the case of shrub lands, Pakistan and India showed a positive trend whereas Sri Lanka, Timor Leste and Myanmar showed a negative trend. In the case of FRP, positive trends were found for forests in the Philippines and grasslands in Thailand. For rest of the countries, FRP trends in different land cover types were not significant.

### VIIRS fire and FRP trends in different countries

Except for the Philippines which showed an increasing trend in fires as well as FRP, for all other countries, the VIIRS fire data trends were not significant. We attribute the insignificant statistical trends from VIIRS to short period (2012–2016) of available data (Supplementary Material).

### VIIRS fire and FRP trends in different land cover types

VIIRS data did not show significant trends at a country level as well as across different land cover types. However, we found a decreasing trend in fires for Laos croplands, an increasing trend in Thailand forests and a decreasing trend in Afghanistan grasslands (Fig. 3; Supplementary Material). FRP in Philippines showed significant positive trends for forests whereas Thailand showed increasing trends in FRP for grasslands (Supplementary Material). Additional years of data are needed to assess trends using VIIRS.

### Anomalies in fires

**Figure 5:**
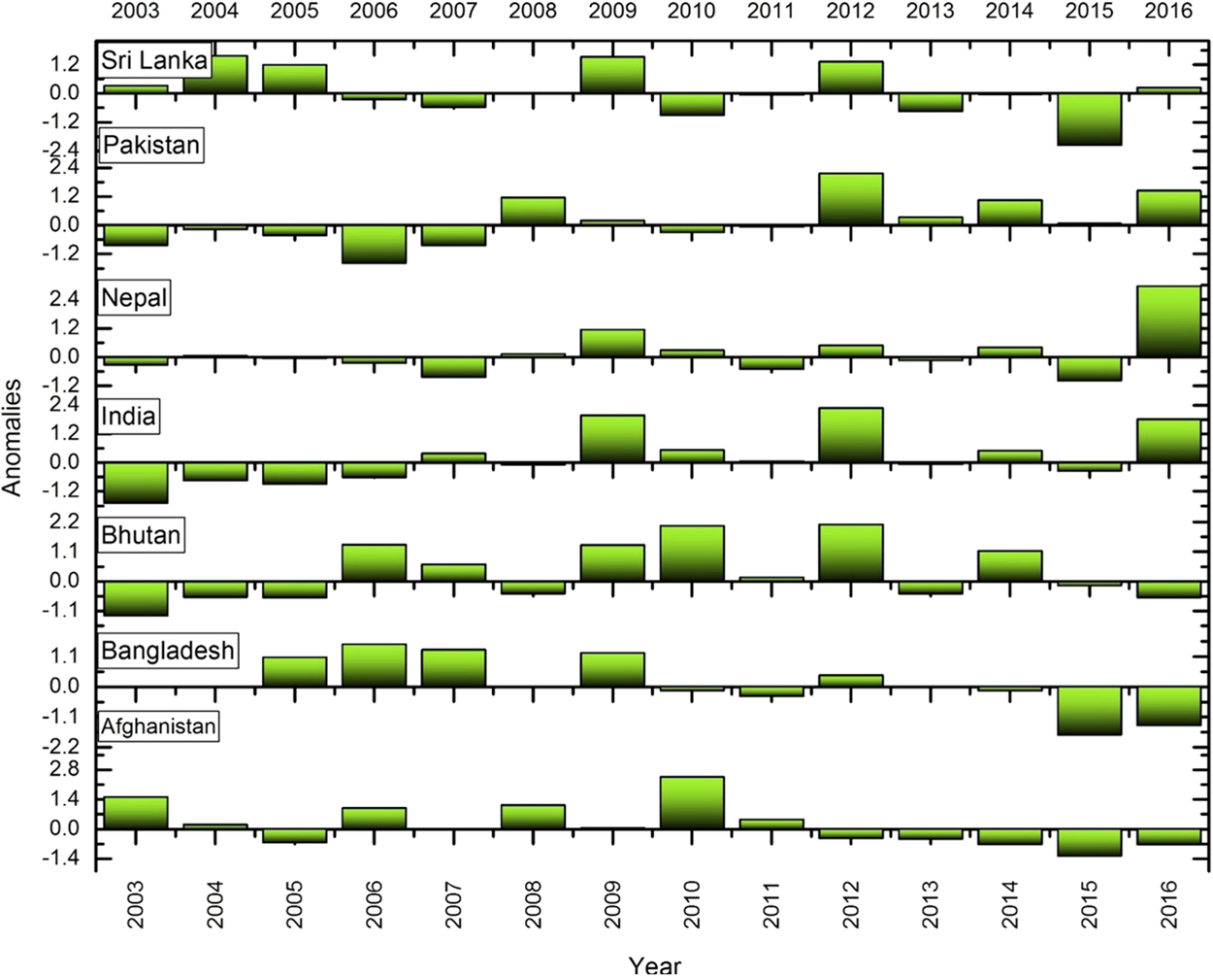
Standardized fire anomalies in South Asia. A positive anomaly means that the fires were higher in a specific year than normal and a negative anomaly indicates that the lesser fires than normal for the specific year.

**Figure 6:**
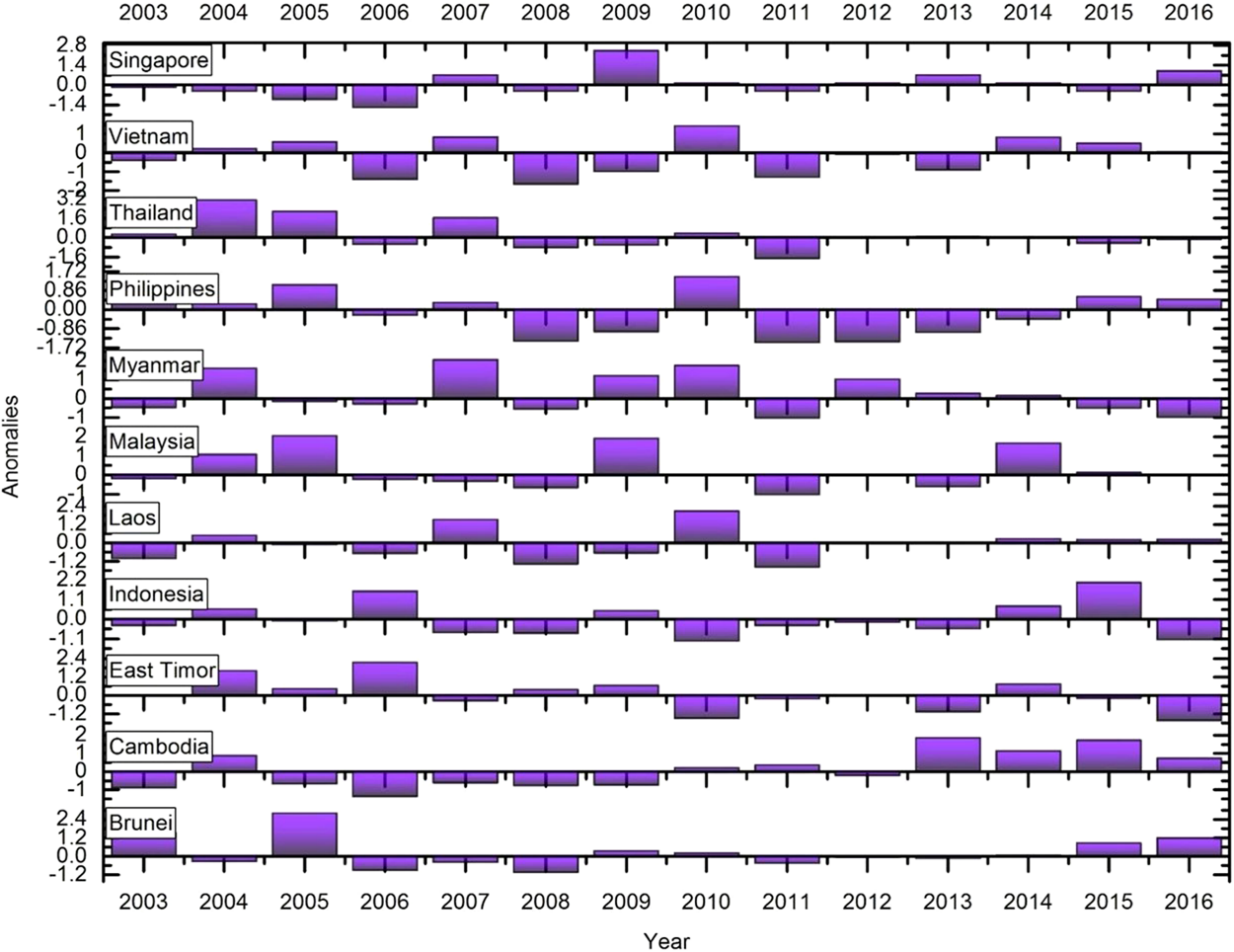
Standardized fire anomalies in Southeast Asia. A positive anomaly means that the fires were higher in a specific year than normal and a negative anomaly indicates that the lesser fires than normal for the specific year.

We used fire anomalies, i.e., the departure from an average value of fire counts over a fourteen-year time in different countries to capture fire variations. A positive anomaly means that the fires were higher in a specific year than normal and a negative anomaly indicates fewer fires than normal for the specific year. Figures 5, 6 depicts the anomalous years where fires are profoundly low. For example, in India, relatively high positive anomalies were found during 2009, 2012 and 2016 whereas negative anomalies are seen during 2003. In Cambodia, high positive anomalies are found during 2013 and 2015. The anomaly statistics can be used to infer drivers of fires. In the case of Indonesia, the positive fire anomalies found during 2006 and 2015 (Fig. 6) can be related to the El Nino induced droughts and positive dipole^6,63,64^. El Nino is associated with an abnormal warming of surface waters in the east and central equatorial Pacific which negatively impacts the monsoon, whereas, La Nina results in abnormal cooling of waters aiding monsoon. However, the influence of El Nino/La Nina can be inconsistent. For example, the El Nino years include 2002, 2004, 2009 and 2015; except for 2009, which showed a significant positive anomaly with higher number of fires, 2004 and 2015 had negative fire anomalies. Thus, relating El Nino events to fire events should be done cautiously. In addition to the anomalies, we also performed an in-depth analysis to explore the relationships between mean monthly fires, temperate and precipitation.

### Climate-precipitation relationships

**Figure 7:**
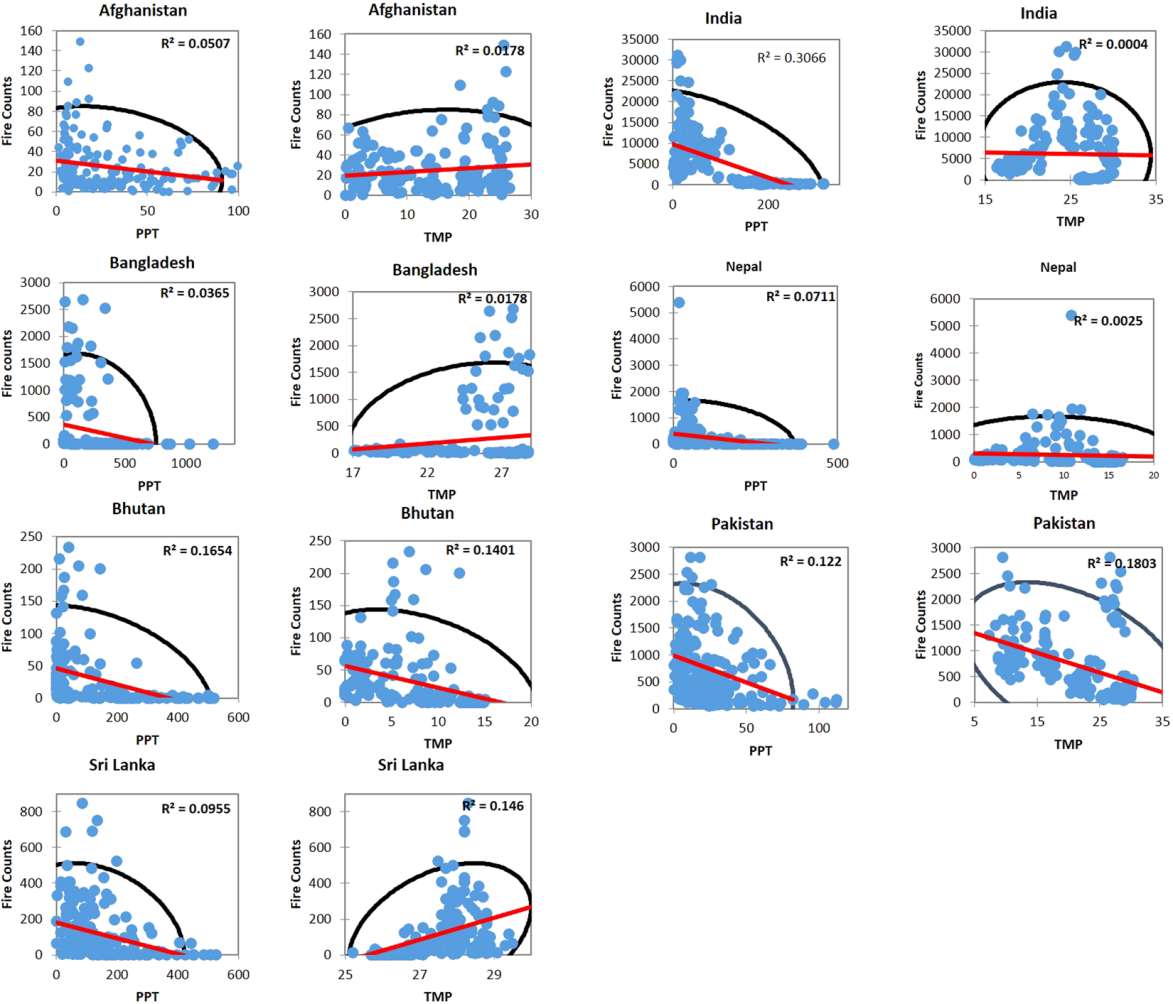
The relationship between monthly fires, precipitation (PPT) and temperature (TMP) in South Asia countries. The blue dots represent MODIS fires, black ellipse correspond to a 95% confidence interval whereas the red line represents the regression line. The r^2^ value also given in the plot.

**Figure 8:**
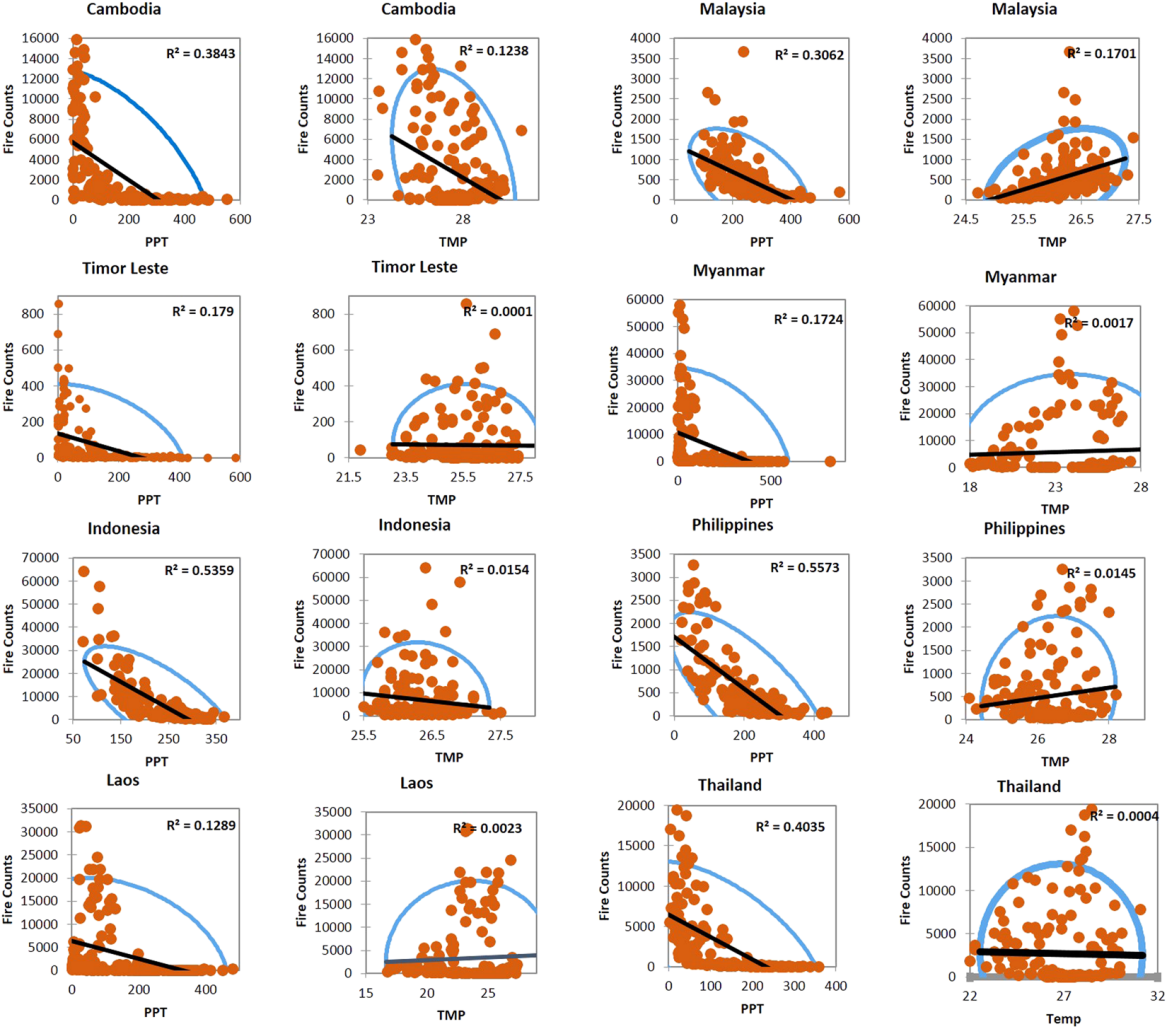
The relationship between monthly fires, precipitation, and temperature in Southeast Asia countries. The orange dots represent MODIS fires, blue ellipse correspond to a 95% confidence interval whereas the black line represents the regression line. The r^2^ value also given in the plot.

**Figure 9:**
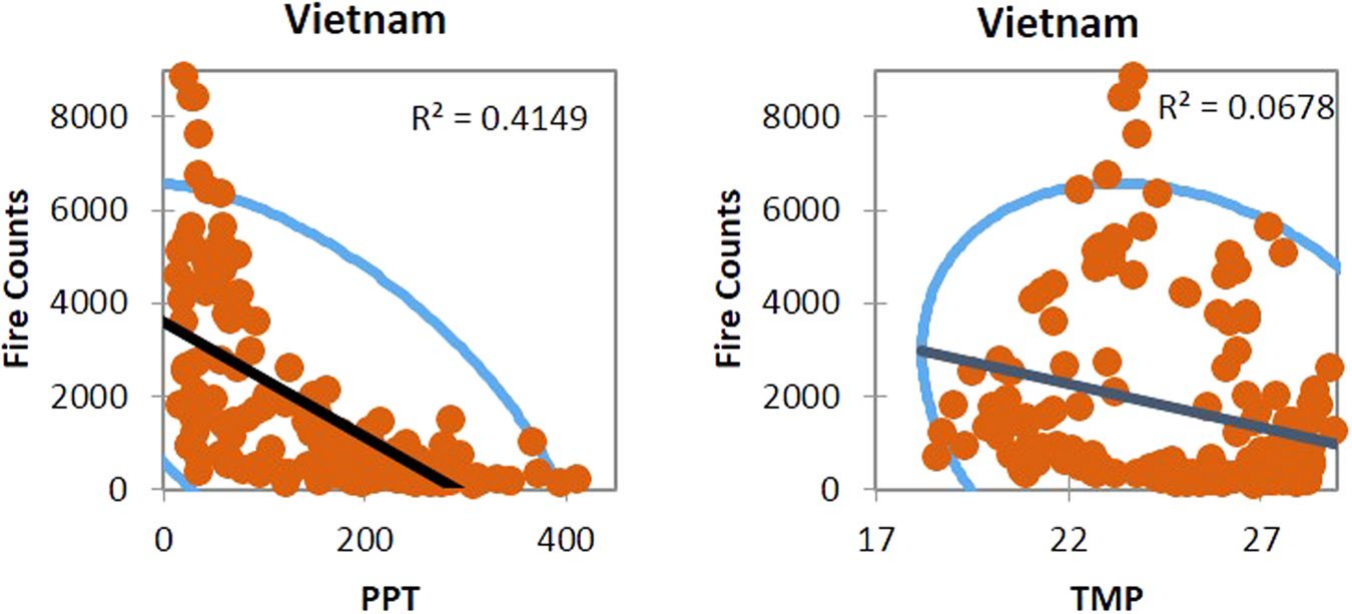
The relationship between monthly fires, precipitation, and temperature for Vietnam. The orange dots represent MODIS fires, blue ellipse correspond to a 95% confidence interval whereas the black line represents the regression line. The r^2^ value also given in the plot.

The relationship between monthly fires, precipitation, and temperature in South and South East Asian countries is shown in Figs 7, 8 and 9. Most of the countries showed a negative slope with decrease in fire counts with an increase in precipitation, however, the r^2^ values were not strong except for India and Bhutan where precipitation could explain 30% and 16% of the variation in fires. Mean monthly temperature showed poor correlations and, in some cases, had no relationship as in Nepal or negative slope as in Pakistan.

Relatively, in Southeast Asian countries, precipitation showed strong negative correlations with fire counts. For example, in the Philippines, 55% of variations in fires could be explained by the precipitation; similarly, 53% in Indonesia, 40% in Thailand, 41% in Vietnam, 38% percent in Cambodia, and 17% in Timor Leste. However, similar to South Asian countries, correlations between the fire counts and temperature were poor. Our results highlighting monthly precipitation as an essential driver of fires is consistent with similar results reported in the literature^35,65,66^. The results reported in this study can contribute to a better characterization of fires based on precipitation and feeding into fuel-behavior models of fire suppression.

In contrast to the climate drivers, our literature review suggests that most of the fires in S/SEA are anthropogenic. A variety of reasons have been documented for example, forests are set on fire for inducing the growth of new grass for grazing^1^, clearing the land for agriculture such as through slash and burn^20,67–70^, for collecting minor forest produce such as honey, edible and non-edible oil seeds, and flowers, to hunt wild animals^71^, in addition to crop residue burning in agricultural landscapes^27,71^. Incorporating these drivers for predicting fire risk in different landscapes of S/SEA countries is a challenging task. Since some of the countries in S/SEA still hold precious forests, protecting them from further fire disasters should be a higher priority for conservation.

## Conclusions

Fire management in S/SEA countries is an increasingly complex and challenging problem that requires detailed information about the occurrence, geographic variation, trends, anomalies, and main drivers of fire within the region. Our current study provides a comprehensive analysis of the above metrics. In South Asia, India had the highest number of annual fires followed by Pakistan and others whereas in Southeast Asia, Indonesia had the highest followed by Myanmar, Laos, etc. Frequency analysis was useful to identify hotspots of grid cells having recurrent fires. Frequency analysis suggested nearly 30.5% of South/Southeast Asia with recurrent fires every year within a fifteen year time period with highest percentage in Laos (95.82%), Cambodia (78.7%), Thailand (75.0%), and Myanmar (75.1%). The frequency data can be integrated into emission models to address biomass burning impacts including transboundary air pollution. Of the different countries, increasing trend in fires were found for India (p = 0.004), Cambodia (p = 0.001), and Vietnam (p = 0.050) and decreasing trend for Timor Leste (p = 0.004). In case of FRP, Afghanistan and Sri Lanka had a negative slope value of 0.04 and 0.049 whereas Cambodia, India, and Pakistan had a positive slope with highly significant (*p*) values.

Partitioning of fires into different land cover categories suggested agricultural fires are as equally recurrent as forest fires, requiring immediate attention specifically in India (p = 0.006), Pakistan (p = 0.047), and Vietnam (p = 0.004) where they are increasing. Agricultural fires can be controlled through stringent and enforced policies and by promoting incorporation of residues into the soil rather than burning. Specific to forest fires, Nepal (82.84%) and Bhutan (75.56%) had the highest percentage of fires in forest land cover types which are mostly human initiated. In the hilly regions, human dependence on forests is significant, thus developing sustainable forest management plans involving local people (community based fire management) should be the priority. Also, our results suggest increasing shrub land fires in India (p = 0.054) and Pakistan (p = 0.073) and we attribute these to forest degradation and fragmentation.

We also characterized anomalies in fires. In India, relatively high positive anomalies were found during 2009, 2012 and 2016 whereas negative anomalies during 2003. For Cambodia, high positive anomalies are found during 2013 and 2015. The anomaly statistics can be used to infer drivers of fires. Also, our results suggests precipitation as an important fire suppressing agent in Southeast Asian countries compared to the South Asian countries. For example, in the Philippines, 55% of variations in fires could be explained by the precipitation; similarly, 53% in Indonesia, 40% in Thailand, 41% in Vietnam, 38% percent in Cambodia, and 17% in Timor Leste. Thus, integrating precipitation data into fire-behavior models for quantifying fire risk can yield promising results. Collectively, our findings highlight essential fire metrics useful for fire management and mitigation in S/SEA countries.

## Supplementary information

Supplementary Data 1

## Data Availability

All data and materials used the study are open access. We will be glad to share the same freely on request.
