# Supplementary material for: Trends in Vegetation fires in South and Southeast Asian Countries
Source: Sci Rep. 2019 May 15;9:7422. doi: 10.1038/s41598-019-43940-x (PMC6520341; doi:10.1038/s41598-019-43940-x)
Supplement: Supplementary file 1 — Supplementary Data 1 [file 41598_2019_43940_MOESM1_ESM.pdf]

Supplementary Material

Trends in Vegetation fires in South and Southeast Asian Countries

Krishna Prasad Vadrevu*, Kristofer Lasko+, Louis Giglio[#], Wilfrid Schroeder^, Sumalika Biswas[&], and Chris Justice[#]

*NASA Marshall Space Flight Center, Huntsville, Alabama, 35811, USA.
+Geospatial Research Lab, US Army Corps of Engineers, Alexandria, Virginia, 22315, USA.
^NOAA NESDIS, College Park, Maryland, 20740, USA.
[&]Smithsonian Conservation Biology Institute, Front Royal, Virginia, 22630, USA.
[#]University of Maryland, College Park, Maryland, 20742, USA.

Table 4. Trends in MODIS Fires (Aqua and Terra Combined) for different Land Cover Types (2003-2016)

Country	Seasonal Kendall Test Fires				Slope Estimator Fires			
	**Croplands**	**Forests**	**Grasslands**	**Shrublands**	**Croplands**	**Forests**	**Grassland**	**Shrublands**
**Cambodia**	1 (0.988)	461.0 (0.000)	412 (0.006)	401 (0.000)	0	13.50	0.667	5
**Indonesia**	-134 (0.219)	80.0 (0.467)	105 (0.065)	115 (0.249)	-18.64	19.75	2.19	4.58
**Laos**	26 (0.752)	-17.0 (0.761)	58 (0.184)	-30.0 (0.512)	0	0	0	0
**Malaysia**	-331.0 (0.008)	17 (0.827)	137 (0.133)	42 (0.743)	-5.5	0.348	0	0.167
**Maldives**	0	0	-135 (0.014)	0	0	0	0	0
**Myanmar**	78 (0.330)	-57.0 (0.336)	27 (0.701)	-117 (0.028)	0	-0.134	0	-0.16
**Philippines**	-111.0 (0.212)	-114 (0.275)	7.0 (0.897)	228 (0.130)	-1.2	-0.817	228	0
**Thailand**	-125 (0.142)	-159.0 (0.03)	-57 (0.308)	-100.0 (0.263)	-3.0	-0.76	0	-0.286
**Timor Leste**	-247.0 (0.022)	-154.0 (0.096)	54 (0.034)	-170.0 (0.002)	-0.250	0	0	-0.091
**Vietnam**	214 (0.004)	76.0 (0.219)	-86.0 (0.145)	19.0 (0.736)	6	3.50	0	0.50
**Afghanistan**	-99 (0.111)	-124 (0.017)	-3.0 (0.976)	-79.0 (0.342)	0	0	0	0
**Bangladesh**	120 (0.027)	-27 (0.566)	169 (0.028)	-35 (0.457)	0	0	0	0
**Bhutan**	94 (0.018)	2 (0.970)	-50 (0.795)	15 (0.627)	0	0	0	0
**India**	255 (0.006)	-6 (0.914)	298 (0.004)	107.0 (0.054)	23.25	0	3.586	0.652
**Nepal**	112 (0.170)	8 (0.857)	-148 (0.037)	-46.0 (0.372)	0	0	0	0
**Pakistan**	161 (0.047)	115 (0.041)	172 (0.034)	101 (0.073)	4.0	0.250	0.732	0.333
**Sri Lanka**	-133 (0.079)	-156 (0.054)	-69 (0.035)	-107 (0.049)	-0.33	-5.66	0	0

Table 5. Trends in MODIS Fire Radiative Power (FRP) (Aqua and Terra Combined) for different Land Cover Types(2003-2016)

Country	Seasonal Kendall Test<br>Fire radiative power				Slope Estimator<br>Fire radiative power			
	Croplands	Forests	Grasslands	Shrublands	Croplands	Forests	Grassland	Shrublands
**Cambodia**	-21.0 (0.761)	392.0 (0.004)	411 (0.000)	363 (0.007)	-1.793	204.34	14.775	98.86
**Indonesia**	-152 (0.168)	0 (1.0)	70.0 (0.505)	114 (0.255)	-625	-0.173	63.43	119.46
**Laos**	-20.0 (0.799)	15 (0.780)	54 (0.308)	-14.0 (0.712)	0	0	0	0
**Malaysia**	-304 (0.014)	44.0 (0.479)	78 (0.352)	104 (0.067)	-136.78	28.49	0.545	14.46
**Maldives**	0	0	-146 (0.068)	0	0	0	-1.0	0
**Myanmar**	50.0 (0.378)	-72 (0.202)	-9.0 (0.901)	-143 (0.011)	4.8	-2.0	0	-3.73
**Philippines**	-106.0 (0.062)	-121.0 (0.262)	23.0 (0.769)	216 (0.156)	-14.99	-12.426	0	72.15
**Thailand**	-136.0 (0.107)	-150.0 (0.04)	-75 (0.259)	-93.0 (0.295)	-52.56	-11.08	-0.867	-3.90
**Timor Leste**	-260 (0.017)	-182 (0.051)	42.0 (0.092)	-180.0 (0.028)	-2.855	-0.797	0	-1.838
**Vietnam**	94.0 (0.166)	48.0 (0.389)	-72.0 (0.219)	14.0 (0.801)	49.63	55.04	-0.709	10.044
**Afghanistan**	-138 (0.014)	-115 (0.028)	-59 (0.572)	-70 (0.362)	-2.175	0	-2.490	0
**Bangladesh**	91 (0.097)	-51 (0.285)	179 (0.025)	-78.0 (0.152)	0	0	0	0
**Bhutan**	84 (0.065)	-16 (0.820)	-39 (0.357)	17 (0.630)	0	0	0	0
**India**	216 (0.006)	-98.0 (0.100)	228 (0.013)	-50 (0.176)	328.75	-25.37	40.2	-8.60
**Nepal**	92 (0.097)	4 (0.939)	-150 (0.019)	-16.0 (0.758)	0.679	0	-1.20	0
**Pakistan**	132 (0.020)	41.0 (0.567)	116 (0.145)	86.0 (0.287)	68.33	1.03	10.681	5.45
**Sri Lanka**	-156.0 (0.005)	-28.0 (0.613)	-71(0.022)	-106 (0.199)	-5.66	0	0	-0.50

Table 6. Trends in VIIRS Active Fires for different Countries (2003-2016)

Country	FC Seasonal Kendall Test	FC Slope Estimator	FRP Seasonal Kendall Test	FRP Slope Estimator
**Cambodia**	0.0 (1.0)	0.333	14.0 (0.447)	175.45
**Indonesia**	-8.0 (0.730)	-353.75	-14.0 (0.544)	-4679.27
**Laos**	6.0 (0.591)	3.125	-4.0 (0.804)	-7.07
**Malaysia**	4.0 (0.822)	21.50	-8.0 (0.586)	-355.583
**Maldives**				
**Myanmar**	10 (0.629)	2.54	2.0 (0.919)	3.73
**Philippines**	38.0 **(0.009)**	114.0	42.0 **(0.054)**	703.50
**Thailand**	22.0 (0.139)	91.33	16.0 (0.136)	345.53
**Timor Leste**	-5.0 (0.777)	-0.833	-12.0 (0.757)	-4.80
**Vietnam**	24.0 (0.22)	148.41	24.0 (0.247)	1300
**Afghanistan**	-20.0 (0.320)	-7.25	-6.0 (0.691)	-3.30
**Bangladesh**	-3.0 (0.887)	-0.16	0 (1.0)	-0.167
**Bhutan**	-13.0 (0.484)	-0.583	-20.0 (0.153)	-4.4
**India**	16.0 (0.370)	266.0	10.0 (0.605)	727.4
**Nepal**	7 (0.701)	0	17.0 (0.340)	2.925
**Pakistan**	17.0 (0.257)	44.79	12 (0.606)	162.06
**Sri Lanka**	1 (1.0)	0.167	-10.0 (0.749)	-18.8

Table 7. Trends in VIIRS Active Fires for different Land Cover Types in South/Southeast Asian Countries (2012-2016)

Country	Seasonal Kendall Test Fires				Slope Estimator Fires			
	Croplands	Forests	Grasslands	Shrublands	Croplands	Forests	Grassland	Shrublands
**Cambodia**	4 (0.851)	16 (0.252)	-55.0 (0.096)	4.0 (0.817)	0.417	0.5	-0.4	0.167
**Indonesia**	-10.0 (0.525)	-6.0 (0.811)	8.0 (0.788)	-26.0 (0.323)	-121.12	-145.25	16.58	-95.25
**Laos**	-26.0 **(0.066)**	21.0 (0.222)	-4.0 (0.876)	2.0 (0.903)	-2.750	3.0	0.0	0.833
**Malaysia**	10.0 (0.638)	-2.0 (0.944)	0 (1.0)	-13.0 (0.58)	11.50	-1.750	0	25.44
**Myanmar**	4.0 (0.832)	-1 (1.0)	5 (0.731)	-16.0 (0.363)	9.30	0	0	-2.58
**Philippines**	33.0 (0.090)	48.0 (0.085)	-18.00 (0.292)	38.0 (0.099)	35.50	44.0	0.708	19.25
**Thailand**	8.0 (0.621)	44.0 **(0.053)**	29.0 (0.133)	33.0 (0.077)	45.125	14.97	2.667	3.5
**Timor Leste**	-6.0 (0.889)	-3.0 (0.943)	-6.0 (0.627)	-14.0 (0.632)	-0.458	0.0	0	0
**Vietnam**	26.0 (0.264)	24.0 (0.175)	15.0 (0.278)	18.0 (0.318)	37.87	104.66	0.375	27.25
**Afghanistan**	4.0 (0.851)	16.0 (0.252)	-55.0 **(0)**	4.0 (0.817)	0.417	0.50	-4.0	0.167
**Bangladesh**	-1.0 (1.0)	-18.0 (0.33)	-8.0 (0.46)	-4.0 (0.779)	-0.167	0	0	0
**Bhutan**	-9.0 (0.574)	-15.0 (0.430)	0 (1.0)	-7.0 (0.66)	0	-0.125	0	0
**India**	16.0 (0.505)	28.0 (0.177)	14.0 (0.443)	22.0 (0.243)	131.91	61.5	17.83	12.0
**Nepal**	17.0 (0.340)	10.0 (0.581)	16.0 (0.456)	23.0 (0.235)	2.925	0	0	0
**Pakistan**	12.0 (0.606)	12.0 (0.637)	23.0 (0.119)	10.0 (0.398)	162.65	207	13.3	20.30
**Sri Lanka**	-8.0 (0.753)	-6.0 (0.875)	0.0 (1.0)	-4.0 (0.905)	-1.5	-0.667	0.0	-0.125

Table 8. Trends in VIIRS Fire Radiative Power (FRP) for different Land Cover Types in South/Southeast Asian Countries (2012-2016)

Country	Seasonal Kendall Test Fire radiative power				Slope Estimator Fire radiative power			
	Croplands	Forests	Grasslands	Shrublands	Croplands	Forests	Grassland	Shrublands
**Cambodia**	4 (0.862)	-1 (1.0)	-10.0 (0.515)	-4.0 (0.832)	2.425	0	-8.8	-0.763
**Indonesia**	-16.0 (0.475)	-10.0 (0.665)	-10.0 (0.731)	-30.0 (0.245)	-813.18	1519.32	-154.42	-667.85
**Laos**	-14.0 (0.211)	6.0 (0.728)	-8.0 (0.765)	-12.0 (0.275)	-7.250	5.03	0.0	-10.625
**Malaysia**	-6.0 (0.667)	-10.0 (0.433)	3.0 (0.941)	-20.0 (0.379)	-34.55	-333.3	0.187	-25.42
**Myanmar**	4 (0.855)	-10.0 (0.525)	-1.0 (1.0)	-12.0 (0.359)	9.30	-2.75	0	-14.0
**Philippines**	32.0 (0.120)	66.0 (0.048)	-14.0 (0.366)	36.0 (0.104)	217.11	249.16	-2.958	106.65
**Thailand**	6.0 (0.676)	22.0 (0.128)	33.0 (0.057)	12.0 (0.368)	99.85	49.27	23.63	19.45
**Timor Leste**	-12.0 (0.760)	-5.0 (0.888)	-11.0 (0.379)	-9.0 (0.765)	-4.88	-0.650	0	0
**Vietnam**	26 (0.281)	26.0 (0.198)	20. (0.288)	14.0 (0.389)	256	580	2.05	234.16
**Afghanistan**	4.0 (0.863)	-1.0 (1.0)	-10.0 (0.515)	-4.0 (0.840)	2.425	0	-8.87	-0.763
**Bangladesh**	16.0 (0.272)	-18.0 (0.330)	-4.0 (0.793)	-4.0 (0.779)	2.917	0	0	0
**Bhutan**	-18.0 (0.224)	-18.0 (0.144)	-10.0 (0.365)	-10.0 (0.158)	-0.350	-3.2	0	0
**India**	-16.0 (0.496)	22.0 (0.157)	26.0 (0.179)	12.0 (0.248)	498.205	173	72.1254	34.44
**Nepal**	10.0 (0.639)	9.0 (0.637)	26.0 (0.224)	24.0 (0.212)	0.229	0.0	0.050	0.0
**Pakistan**	12.0 (0.637)	20.0 (0.361)	26.0 (0.218)	20.0 (0.179)	207.3	7.0	49.75	4.0
**Sri Lanka**	-26.0 (0.346)	0.0 (1.0)	-1.0 (1.0)	-10.0 (5.165)	-22.0	0.60	0.0	-2.06
